# Frequent, Short Physical Activity Breaks Reduce Prefrontal Cortex Activation but Preserve Working Memory in Middle-Aged Adults: ABBaH Study

**DOI:** 10.3389/fnhum.2021.719509

**Published:** 2021-09-16

**Authors:** Emerald G. Heiland, Olga Tarassova, Maria Fernström, Coralie English, Örjan Ekblom, Maria M. Ekblom

**Affiliations:** ^1^Department of Physical Activity and Health, The Swedish School of Sport and Health Sciences (GIH), Stockholm, Sweden; ^2^Department of Physiology, Nutrition, and Biomechanics, The Swedish School of Sport and Health Sciences (GIH), Stockholm, Sweden; ^3^School of Health Sciences and Priority Research Centre for Stroke and Brain Injury, University of Newcastle, Callaghan, NSW, Australia; ^4^Centre for Research Excellence in Stroke Recovery and Rehabilitation, Florey Institute of Neuroscience and Hunter Medical Research Institute, Callaghan, NSW, Australia; ^5^Department of Neuroscience, Karolinska Institutet, Solna, Sweden

**Keywords:** cerebral blood flow, cognition, functional near-infrared spectroscopy, exercise, sedentary

## Abstract

Prolonged sitting is increasingly common and may possibly be unfavorable for cognitive function and mood. In this randomized crossover study, the effects of frequent, short physical activity breaks during prolonged sitting on cognitive task-related activation of the prefrontal cortex were investigated. The effects on working memory, psychological factors, and blood glucose were also examined, and whether arterial stiffness moderated prefrontal cortex activation. Thirteen subjects (mean age 50.5 years; eight men) underwent three 3-h sitting conditions, interrupted every 30-min by a different 3-min break on separate, randomized-ordered days: seated social interactions (SOCIAL), walking (WALK), or simple resistance activities (SRA). Arterial stiffness was assessed at baseline. Before and after each 3-h condition, psychological factors (stress, mood, sleepiness, and alertness) were assessed through questionnaires and functional near-infrared spectroscopy (fNIRS) was used to measure changes in prefrontal oxygenated hemoglobin (Oxy-Hb), indicative of cortical activation, while performing working memory tasks [1- (baseline), 2-, and 3-back]. Blood glucose levels were continuously measured throughout the conditions. Results revealed no significant changes in Oxy-Hb during the 2-back compared with the 1-back test in any condition, and no time-by-condition interactions. During the 3-back test, there was a significant decrease in Oxy-Hb compared with the 1-back after the WALK condition in the right prefrontal cortex, but there were no time-by-condition interactions, although 3-back reaction time improved only in the WALK condition. Mood and alertness improved after the WALK condition, which was significantly different from the SOCIAL condition. Arterial stiffness moderated the effects, such that changes in Oxy-Hb were significantly different between WALK and SOCIAL conditions only among those with low arterial stiffness. Blood glucose during the interventions did not differ between conditions. Thus, breaking up prolonged sitting with frequent, short physical activity breaks may reduce right prefrontal cortex activation, with improvements in some aspects of working memory, mood, and alertness.

**Clinical Trial Registration:**www.ClinicalTrials.gov, identifier NCT04137211.

## Introduction

Sedentary behavior (defined as an energy expenditure of <1.5 METs while lying, sitting, or reclining, while awake (Tremblay et al., 2017)) can be detrimental for cognitive performance (Falck et al., 2017), whereas acute physical activity breaks may elicit positive effects (Carter et al., 2018). One of the main underlying mechanisms driving the acute physical activity-induced improvements on cognitive performance is assumed to be changes in cerebral blood flow driven by neural activation (Pontifex et al., 2019; Chandrasekaran et al., 2021). Previous research findings have demonstrated that a single bout of physical activity (about 10–20 min) can increase such task-related cerebral blood flow, with coinciding improvements in prefrontal cortex-dependent cognitive tasks (Herold et al., 2018). However, decreases in task-related cerebral blood flow after an exercise bout with concomitant enhancement in cognitive performance has also been observed (Murata et al., 2015; Moriarty et al., 2019). Thus, more studies are needed to understand the effects of acute physical activity on neural task-related changes in cerebral blood flow as a measure of cortical activation.

Moreover, the effects of a single bout of 10–20 min exercise of moderate-to-vigorous intensity on cognitive and psychological well-being are unlikely to compensate for a whole day of sitting. Frequent (every 20–30 min), short (approximately 1–3 min) physical activity breaks throughout the workday may be a more feasible strategy to offset the negative effects of prolonged sitting on cognitive performance and cerebral blood flow. Indeed, these types of breaks have been shown to be beneficial for central and peripheral vascular function (Loh et al., 2020), which are also compromised by prolonged sitting, but their effects on cortical activation remain unknown. Resistance exercise breaks may also improve vascular function (Dempsey et al., 2016a, b), which in turn may help in the maintenance of cerebral blood flow and cognitive function by means of reducing glycemic excursions throughout the day (Wheeler et al., 2017). Indeed positive effects have also been seen on executive function from acute resistance exercise (Tsukamoto et al., 2016; Pontifex et al., 2019). Thus, exercise breaks of different intensities may not only benefit vascular function, but also has the potential to improve cerebral blood flow and cognitive function, but requires further investigation.

Previous studies assessing the effects of physical activity breaks on prefrontal cortex activation are limited and inconsistent, mainly due to differing study designs and measurement techniques of cerebral blood flow and cognition. Most earlier studies assessed cerebral blood flow mainly as alterations in blood flow velocity of the middle cerebral artery using transcranial Doppler ultrasound (Carter et al., 2018; Perdomo et al., 2019; Wheeler et al., 2019; Maasakkers et al., 2020), which disregards region-specific and cognitive-task-related changes in the cerebral hemodynamic response, thus eliminating the ability to localize changes in cortical activation. Findings from these studies on more global changes in cerebral blood flow velocity were highly discordant with increases observed in healthy desk workers (Carter et al., 2018), decreases in middle-aged adults with hypertension (Perdomo et al., 2019) and older adults (Wheeler et al., 2019), and no changes in older adults (Maasakkers et al., 2020).

Functional near-infrared spectroscopy (fNIRS) is another portable and non-invasive technique, but with the ability to measure regional task-related cerebral blood flow changes at the cortical level with high temporal resolution (Herold et al., 2018; Pinti et al., 2020). Specifically, fNIRS monitors changes in oxygenated- (Oxy-Hb) and deoxygenated-hemoglobin (d-Oxy-Hb), and is based on the theory of neurovascular coupling. This theory postulates that increased neural demands elicit a corresponding increase in oxygen delivery to meet local energy requirements (Herold et al., 2018). The fNIRS measure of changes in Oxy-Hb has been found to significantly correlate with prefrontal cerebral task-related changes in functional magnetic resonance imaging (fMRI) blood-oxygen-level dependent signals (Sato et al., 2013). Furthermore, fNIRS has a high tolerance to motion artifacts, is of low cost, and is suitable for use in different populations and settings (Herold et al., 2018). These additional advantages can allow for advanced study into the underlying physiological effects of physical activity breaks on prefrontal cortex activation during a working memory task. Yet, fNIRS has not been previously employed in this capacity.

In addition, working memory is an important cognitive function for everyday performance, but inhibitory control has been more prominent in previous studies (Pontifex et al., 2019). Working memory is an important component of the executive functions, encompassing a wide variety of higher-level processes that requires storage and manipulation of information to meet task goals in a short period of time (Baddeley and Hitch, 1994). Studies on region-specific, task-related changes in Oxy-Hb can better elucidate the underlying mechanisms explaining the effects of physical activity breaks on working memory.

Furthermore, the ability to regulate cerebrovascular blood flow from the changing diameter of the vessels is likely to depend on arterial stiffness (Tarumi et al., 2013). Central arterial stiffness has been reported to be lower in well-trained adults compared with their sedentary counterparts (Tanaka et al., 2000). However, whether variation in arterial stiffness can moderate the effects of cortical activation from frequent, short physical activity breaks, is yet to be investigated. Acute exercise and frequent, short bouts of physical activity during prolonged sitting have also been recognized as beneficial for psychological outcomes among young active as well as middle-aged overweight and obese participants (Wennberg et al., 2016; Niedermeier et al., 2020). It is, unknown whether breaking up sitting with short physical activity breaks would also benefit psychological outcomes in healthy middle-aged adults. We designed this study to understand more of how physical activity breaks in prolonged sitting might affect task-related prefrontal cortex activation as a possible mechanism underlying effects on cognition and mental state.

The primary research question of this study was:

1. What are the effects of uninterrupted sitting and frequent, short activity breaks during 3-h of sitting on activity related changes in prefrontal cortex activation measured as Oxy-Hb?

Our secondary questions were:

2. What are the effects of uninterrupted sitting and frequent, short activity breaks during 3-h of sitting on:
  a. Cognitive performance,
  b. Psychological factors (stress, mood, alertness, and sleepiness), and
  c. Post-prandial blood glucose?
3. Does vascular health (arterial stiffness) moderate the effects of activity breaks on Oxy-Hb?

## Materials and Methods

### Design

We conducted a three-condition randomized crossover experimental study. All study procedures took place at the Swedish School of Sport and Health Sciences (GIH) in Stockholm, Sweden. A full description of the study procedures can be found in the protocol paper (Heiland et al., 2020). This trial has obtained ethical approval by the Swedish Ethical Review Authority, Stockholm, Sweden (Dnr 2019-00998). This trial was registered at www.clinicaltrials.gov (NCT04137211) on October 23, 2019, after recruitment began on May 13, 2019. Data collection was completed on March 13, 2020.

### Participants

Adults aged between 40 and 60 years and with a body mass index <35 kg/m^2^ were recruited. Those diagnosed with diabetes, epilepsy, heart failure, stroke, or myocardial infarction, or were receiving current treatment for high blood pressure, sleep disorders, depression, or psychosis, were excluded.

### Familiarization Session

Participants attended an initial visit to the GIH Laboratory that included collecting demographic data, fitness testing (an incremental treadmill test) (see Supplementary Material), providing general health information (questionnaires), being acquainted with experimental procedures, and practicing of the cognitive tests. At the end of this visit, participants were assigned the random order for their conditions. Randomization blinded to the study staff was not possible in this type of study design, but not considered to affect the outcomes. Demographic data included were age, sex, height, weight, and head circumference (to determine the optimal fNIRS cap size).

### Pre-condition Monitoring

Time spent in physical activity and sleep during the 24 h period prior to each condition, was measured subjectively through standardized diaries and objectively *via* worn activity monitors (physical activity: hip-worn actiGraph GT3X+ and activPAL micro; sleep: wrist-worn actiGraph GT3X+).

Dietary intake for the 24 h prior to each condition was recorded in a standardized food diary and closely matched across each pre-condition day. Blood glucose monitors were worn during the 24 h prior to, and during each experimental condition, with checks performed upon arrival at the laboratory, after the standardized breakfast, and at the end of the 3-h intervention.

### Experimental Conditions

**FIGURE 1:**
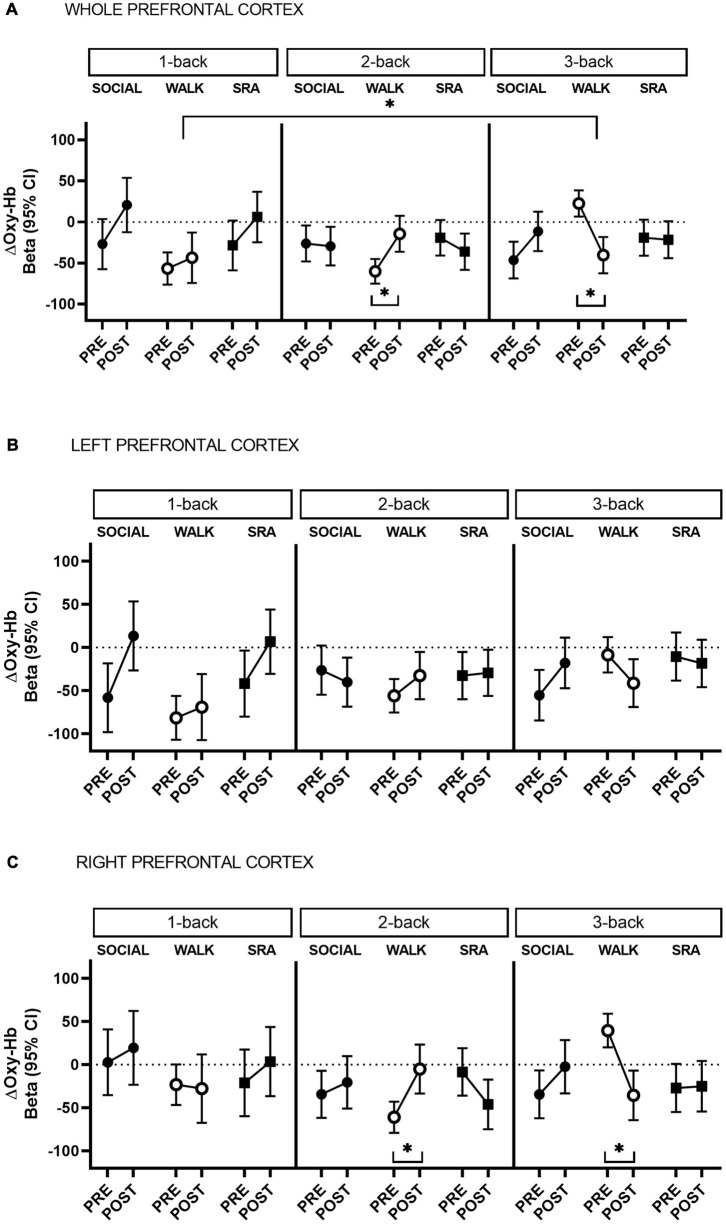
Change in oxygenated hemoglobin (Oxy-Hb) during 1-back, 2-back, and 3-back cognitive tasks from pre-test to post-test, in the whole prefrontal cortex **(A)** and separated by left **(B)** and right **(C)** hemisphere. CI, confidence interval; SOCIAL, social break condition; WALK, walking break condition; SRA, simple resistance activity break condition. *Significant differences within conditions or compared to the baseline (1-back), *q*-values ≤ 0.05 (FDR-adjusted *p*-values for multiple comparisons).

The three experimental conditions, which took place at the same time each visit, and had a minimum 4-day washout between each condition, were: (A) 3-h uninterrupted sitting with 3-min social breaks every 30 min (SOCIAL); (B) 3-h sitting with 3-min bouts of moderate-intensity walking on a treadmill every 30 min (WALK); and (C) 3-h sitting with 3-min bouts of simple resistance activities every 30 min (SRA) [see Figure 1 in the protocol article (Heiland et al., 2020)]. The SOCIAL break (condition A) consisted of a 3-min chat between a research staff and the participant. The WALK break (condition B) was performed at moderate intensity [75–80% of maximal heart rate (Heiland et al., 2020)]. The SRA break (condition C), involved following a standardized video with light body weight exercises (i.e., three rounds of seven half-squats, nine calf raises, six alternating knee raises with gluteal contractions after each knee raise). While sitting, participants were permitted to read a book and drink water, but not allowed to use any technological devices. They were also encouraged to perform the same activity while sitting at each visit. At 135 min into the experimental condition, participants were offered a toilet break.

On the morning of testing, participants provided four saliva samples (for cortisol testing) – immediately after waking and at 30, 45, and 60 min thereafter. Upon arrival at the laboratory, pre-condition outcome measures were collected, followed by an individually standardized breakfast.

### Sample Size

As published in the protocol article (Heiland et al., 2020), effect sizes from previous studies (Hyodo et al., 2012; Endo et al., 2013; Byun et al., 2014; Bediz et al., 2016; Carter et al., 2018; Kujach et al., 2018; Ichinose et al., 2020) using G^∗^Power software (Franz Faul, Universität Kiel, Germany, v 3.1.9.2) were calculated using a *post hoc*, two-tailed, *T*-test of mean differences of matched pairs. Results showed effect sizes between 0.9 and 2.4 for changes in Oxy-Hb. In a subsequent *a priori* analysis using the aforementioned effect sizes, sample sizes calculated ranged between 6 and 13 individuals with an α = 0.05 and β = 0.8 assuming a two-tailed test. Thirteen subjects were chosen based on the largest sample size.

### Outcome Measures

Outcome measures were collected immediately prior to, and immediately after each experimental condition.

#### Oxygenated Hemoglobin

Changes in prefrontal Oxy-Hb and d-Oxy-Hb during working memory tasks ( 1-, 2-, and 3-back tests) were measured using a multi-channel continuous wave functional near-infrared spectroscopy (fNIRS) instrument (portable NIRSport, 8-8 system), with short-separation channels (NIRx Medizintechnik GmbH, Berlin, Germany). Using the NIRx NIRScap, the optodes were positioned over the prefrontal cortex according to the predefined montage with a standard layout. The NIRScap layout for the prefrontal cortex has eight LED light sources and seven detectors placed according to the standard 10–20 system, offering an ensured source-detector separation distance of 3 cm (Supplementary Figure 1). One additional detector was split into eight short-separation detectors placed at a distance of 0.8 cm from each source, to capture superficial blood flow. The cap was placed about 2 cm above the nasion, with the nasion in line with the Fpz position on the cap. A marker was placed below the bottom margin of the cap on the forehead to increase accuracy in repeated replacement of the cap during test days. System calibration was performed before each assessment using NIRStar 15.2 software, and using the predefined montage; and the fNIRS signals were visually quality checked during data collection for motion artifacts. Data were sampled at 7.81 Hz at wavelengths 750 and 820 nm. Changes in Oxy-Hb concentrations were the primary outcome, because it has been shown to be most correlated with regional cerebral blood flow changes (Hoshi et al., 2001). Deoxygenated-hemoglobin concentration changes are reported in Supplementary Material. Further details about system calibration and data processing are found in the protocol article (Heiland et al., 2020).

#### Cognitive Performance

Cognitive performance in working memory was assessed using computerized, numerical N-back tests ( 1-, 2-, and 3-back tests), administered simultaneously with fNIRS measures (Supplementary Figure 2). Initially, each participant was required to stare at a white dot on a black screen for 60 s. Prior to each N-back test, a 40-s practice session with feedback was performed followed by 25 s of rest while staring at the white dot on the screen. Participants were required to indicate (*via* a key press within 2 s from stimulus onset) whether the digit presented on the screen was the same digit as the digit presented 1 stimulus previously (1-back), 2 stimuli previously (2-back), or 3 stimuli previously (3-back). Each digit was presented for 1.5 s at an interstimulus interval of 500 ms. The N-back tests were created using E-Prime 2.0 (Psychology Software Tools, Inc., Pittsburgh, PA, United States). Practice tests were performed at the familiarization session and prior to data collection on each experimental day to reduce learning effects. The outcome variables for cognitive performance included average reaction time (ms) and accuracy (average number of correct responses) for each test across 2 blocks of 20 digit sequences (1-back) or 4 blocks of 20 digit sequences (2- and 3-back). Between each block, instructions were reiterated on the screen until the participant decided to proceed to the next block.

#### Other Measures

Arterial stiffness, defined as the augmentation index (AIx), was measured using SphygmoCor Technology (Nelson et al., 2010; Butlin and Qasem, 2017). After 5 min of supine rest, high fidelity pressure waveforms were recorded (three times). Estimates with an operator index ≥ 75% were averaged to determine the AIx in percentage, and then dichotomized based on the median split to categorize as low (<23.5%) or high (≥23.5%).

Stress was measured using salivary cortisol concentrations using ELISA kit Abcam, ab154996, after being centrifuged at 4 C, at 2800 rpm, for 10 min, and then frozen at −80 C. Three of the saliva samples were used for analysis in this study – the morning sample (immediately after waking up), and pre- and post-intervention testing samples. Additionally, psychological outcomes, including mood [Positive and Negative Affect Scale (PANAS)] (Crawford and Henry, 2004); alertness [10-cm visual analogue scale (VAS)] (Monk, 1989); and sleepiness (Karolinska Sleepiness Scale Questionnaire) (Putilov and Donskaya, 2013) were assessed pre- and post-experimental conditions. Blood glucose was collected continuously, and the area under the curve (AUC) determined during the 3-h intervention period.

### Statistical Analysis

#### fNIRS Data Processing and Analysis

Signals from fNIRS were pre-processed and analyzed using the MATLAB (R2020a, MathWorks, Inc., United States) based software NIRS Brain AnalyzIR Toolbox (Santosa et al., 2018).^1^ Raw voltage data were converted to optical density, followed by estimation of relative changes in hemoglobin state concentrations according to the modified Beer–Lambert law and a partial path-length factor (PPF) of 0.1 (PPF = differential path-length factor/partial volume correction = 5/50 = 0.1). First-level statistics involved examining the evoked signal between each source-detector pair using a general linear model (GLM) with a design matrix constructed for the convolution of the stimulus timing and duration (35 s) with a canonical hemodynamic response function, peaking at 6 s. To correct for motion or systemic physiological confounders an autoregressive pre-whitening approach using iteratively reweighted least-squares (AR-IRLS) (Barker et al., 2013) was employed within the GLM including short-separation channel regressors (Santosa et al., 2020), with no other correction applied to minimize manipulation of the data as suggested by Santosa et al. (2018). This has been deemed the best approach (Santosa et al., 2020). Regression coefficients (betas) and error covariances were solved for in the GLM for each channel in each participant, at each condition, at pre-test and post-test to test statistical differences during each cognitive task in each condition, and between conditions ( 2- and 3-back) and baseline (1-back), in the subject-level statistics. The estimated betas and error covariances in the subject-level statistics for each channel were subsequently used at the group-level statistics.

The group-level statistical models were performed using linear mixed-effects models with condition and time as fixed effects and subject as a random effect to assess within condition differences in the changes in Oxy-Hb (ΔOxy-Hb) from pre- to post-test, averaged over the prefrontal cortex, and separated by right and left hemisphere. The estimated betas from the subject-level analysis from the 1-back, 2-back, and 3-back were employed in the models, and used to test contrasts (baseline vs. 2-back; and baseline vs. 3-back). Time (post–pre) and condition (SOCIAL, WALK, and SRA) interactions were used to estimate intervention effects (between conditions) in the linear mixed-effects models.

A task-based baseline was chosen with minimum arousal for enhanced comparability. The AR-IRLS approach was also chosen to address the issue of serially correlated errors and heavy-tailed noise distributions in fNIRS data (Huppert, 2016). This has been suggested to be the best approach to control for type-I errors in the fNIRS model due to serial correlation (Barker et al., 2013). A false discovery rate (FDR) correction using a Benjamini–Hochberg procedure was used to correct for multiple comparisons, with a critical level of significance set at FDR-adjusted *p* ≤ 0.05 (denoted as *q*-value). Type II power was also reported (Santosa et al., 2018).

#### Analysis of Secondary Outcomes

The modifying effect of baseline arterial stiffness on changes in Oxy-Hb was assessed in additional stratified linear mixed-effects models in the NIRS Brain AnalyzIR Toolbox software using arterial stiffness as a dichotomized variable based on the median split, because of its skewed nature.

For the other secondary outcomes (i.e., cognitive performance, psychological factors, and AUC of glucose), linear mixed-effects models were performed in Stata version 15 (StataCorp, College Station, TX, United States), using subject as a random effect, to investigate within-person changes from pre- to post-test, and time-by-condition interactions to test intervention effects. Linear mixed-effects models, with subject as random effect were also performed to assess within-subject baseline differences. Statistical significance level was set at *p* ≤ 0.05.

## Results

Fifteen people were recruited to the study, but two could not complete all three visits. Overall baseline characteristics of the 13 participants can be found in Table 1, and by condition in Table 2, including accelerometer-derived physical activity and sleep behaviors the day/night before each condition, and differences between conditions. The accelerometer results showed that the time spent sedentary was significantly higher on the day before the WALK condition compared with the day before the SOCIAL condition. In addition, significantly less time was spent in light physical activity on the day before the WALK condition compared with the days before the SOCIAL and SRA conditions.

**TABLE 1:** Means and standard deviations (SD) for baseline characteristics of participants.

	**All (*n* = 13)**
Age, years	50.5 (4.6)
Men, *n* (%)	8 (61.5)
Weight, kg	76.5 (12.8)
Height, m	1.8 (7.3)
Body mass index, kg/m^2^	24.0 (2.4)
Cardiorespiratory fitness, mL/min/kg	46.1 (5.4)

**TABLE 2:** Baseline participant characteristics by condition, and accelerometer-derived physical activity and sleep behaviors the day/night before each condition, and differences between conditions (*n* = 13).

	**Mean or median (SD or IQR)**			**Beta-coefficient (95% confidence interval)**		
	**SOCIAL**	**WALK**	**SRA**	**WALK-SOCIAL**	**SRA-SOCIAL**	**WALK-SRA**
Mean lying systolic blood pressure (SD), mmHg	121.8 (12.6)	119.1 (11.7)	122.9 (13.7)	−2.5 (−6.7, 1.8)	1.2 (−3.1, 5.4)	−3.6 (−7.9, 0.6)
Mean lying diastolic blood pressure (SD), mmHg	74.5 (9.1)	72.2 (9.4)	76.5 (10.2)	−2.4 (−5.8, 1.1)	1.9 (−1.5, 5.4)	−4.3 (−7.8, −0.8)^*^
Mean morning cortisol (SD), ng/ml	6.6 (2.2)	6.8 (3.7)	4.8 (1.8)	0.3 (−1.2, 1.8)	−1.7 (−3.2, −0.2)^*^	2.0 (0.6, 3.4)^*^
Median augmentation index (IQR), %	22.2 (15.4)	25.8 (12.8)	23.6 (12.7)	3.7 (−8.0, 7.3)	−0.3 (−1.3, 3.7)	0.5 (−5.3, 3.7)
Mean heart rate (SD), beats per minute	51.3 (8.0)	51.8 (7.0)	51.2 (8.4)	0.5 (−1.4, 2.5)	−0.1 (−2.0, 1.9)	0.6 (−1.3, 2.5)

**Accelerometer results from day/night before each condition**						

	**Mean (standard deviation)**			**Beta-coefficient (95% confidence interval)**		

Total time of sedentary bouts, min	146.6 (67.0)	235.9 (252.7)	141.4 (89.0)	88.6 (−29.1, 206.3)	−7.0 (−121.8, 107.8)	95.6 (−15.9, 207.1)
% in sedentary	52.1 (9.0)	61.0 (14.5)	56.0 (11.9)	8.5 (1.1, 15.8)^*^	2.8 (−4.3, 9.9)	5.6 (−1.2, 12.5)
% in light	40.3 (9.0)	32.3 (13.0)	38.7 (10.5)	−8.0 (−13.5, −2.5)^*^	−1.0 (−6.3, 4.4)	−7.1 (−12.2, −1.9)^*^
% in moderate	7.3 (2.9)	6.2 (5.2)	5.0 (3.5)	−0.7 (−3.3, 2.0)	−1.9 (−4.5, 0.7)	1.2 (−1.3, 3.7)
% in vigorous	0.3 (0.5)	0.4 (0.6)	0.3 (0.5)	0.1 (−0.2, 0.5)	0.1 (−0.3, 0.4)	0.1 (−0.2, 0.4)
% in MVPA	7.6 (2.9)	6.6 (5.7)	5.3 (3.8)	−0.5 (−3.4, 2.4)	−1.8 (−4.7, 1.0)	1.3 (−1.4, 4.0)
Step counts	9620 (2258)	8537 (4958)	7883 (3914)	−849 (−3666, 1969)	−1426 (−4172, 1321)	577 (−2088, 3242)
Sleep duration, h	7.1 (0.6)	7.2 (0.7)	7.2 (0.8)	0.1 (−0.4, 0.6)	0.1 (−0.4, 0.6)	−0.03 (−0.5, 0.5)

*SOCIAL, social break condition; WALK, walking break condition; SRA, simple resistance activity break condition; MVPA, moderate-to-vigorous physical activity; SD, standard deviation; IQR, Interquartile range.*

*Missing 1 in WALK and SOCIAL for Augmentation index due to operator index < 75%; Missing 2 in SOCIAL and 1 in WALK and SRA for morning cortisol. Missing 3 in SOCIAL, 2 in WALK, and 1 in SRA from accelerometer results.*

^*^Significant difference (*p* ≤ 0.05) between conditions.

### Primary Outcome: Changes in Oxygenated Hemoglobin

During the 2-back in the WALK condition there was a significant increase in Oxy-Hb from pre-test to post-test [Beta 45.7 (95% CI 19.6, 71.8), *q*-value 0.01, Power = 0.82] (Figure 1A). This was not significantly different from the 1-back (baseline) during the WALK condition (Supplementary Table 1). In contrast, there was a decrease in Oxy-Hb during the 3-back test in the WALK condition [Beta −62.9 (95% CI −89.8, −36.0), *q*-value 0.001, Power = 0.98] (Figure 1A), which was significantly different from the baseline (Supplementary Table 1). There were no significant time-by-condition interactions, suggesting that the within condition changes in Oxy-Hb comparing the 2-back and 3-back to the baseline did not differ between conditions.

The within condition changes from pre-test to post-test demonstrated a lateralization. Specifically, the increase in Oxy-Hb during the 2-back in the WALK condition and the decrease in the 3-back were statistically significant only in the right prefrontal cortex [2-back: Beta 55.8 (95% CI 22.8, 88.8), *q*-value 0.02, Power = 0.80; 3-back: Beta −75.2 (95% CI −109.1, −41.2), *q*-value 0.001, Power = 0.97] [Figure 1B (left) and C (right) and Supplementary Table 2]. In comparison to the baseline in the WALK condition, the decrease in Oxy-Hb in the 3-back showed right hemispheric dominance, but became non-significant after correction for multiple comparisons [Beta −70.6 (95% CI −126.5, −14.8), *q*-value 0.08, Power = 0.50] (Supplementary Table 1).

### Secondary Outcome: Moderating Effect of Arterial Stiffness on Oxygenated Hemoglobin

After stratifying by arterial stiffness, across the whole prefrontal cortex, there was a significant increase from pre-test to post-test in Oxy-Hb in those with high arterial stiffness during the 1-back SOCIAL [Beta 71.7 (95% CI 15.4, 127.9), *q*-value 0.04, Power = 0.50; Supplementary Table 3] and 2-back in the WALK condition [Beta 42.2 (95% CI 11.4, 73.0), *q*-value 0.04, Power = 0.60; Supplementary Table 4], and a decrease during the 3-back in the WALK condition [Beta −55.0 (95% CI −86.9, −23.2), *q*-value 0.01, Power = 0.81; Supplementary Table 5]. Among those with low arterial stiffness, in the WALK condition, there was an increase in Oxy-Hb during the 1-back [Beta 79.1 (95% CI 18.2, 140.0), *q*-value 0.04, Power = 0.52; Supplementary Table 3] and 2-back [Beta 92.0 (95% CI 48.6, 135.5), *q*-value 0.002, Power = 0.95; Supplementary Table 4], and a decrease in 3-back [Beta −82.0 (95% CI −126.5, −37.5), *q*-value 0.01, Power = 0.87; Supplementary Table 5]. In addition, in the SRA condition there was a decrease in Oxy-Hb during the 2-back in those with low arterial stiffness [Beta −57.5 (95% CI −102.5, −12.4), *q*-value 0.04, Power = 0.50; Supplementary Table 4].

**FIGURE 2:**
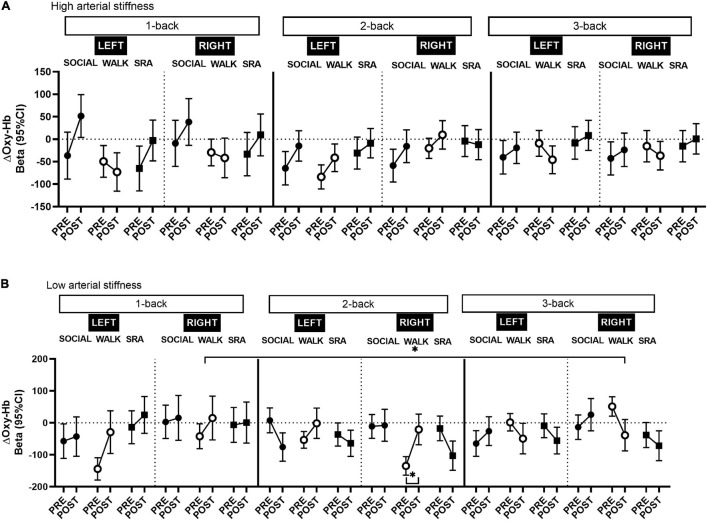
Changes in oxygenated hemoglobin (Oxy-Hb) in the left and right prefrontal cortex among those with **(A)** high arterial stiffness (*n* = 7) and **(B)** low arterial stiffness (*n* = 6), during the 1-back, 2-back, and 3-back tasks from pre-test to post-test. CI, confidence interval; SOCIAL, social break condition; WALK, walking break condition; SRA, simple resistance activity break condition. *Significant differences within conditions or compared to the baseline (1-back), *q*-values ≤ 0.05 (FDR-adjusted *p*-values for multiple comparisons).

In neither the right or left prefrontal cortex were there any significant changes in Oxy-Hb among those with high arterial stiffness (Figure 2A). However, right-lateralized prefrontal cortex activity was observed in those with low arterial stiffness during the 2-back in the WALK condition (Figure 2B and Supplementary Table 4), such that the increase in Oxy-Hb was significant only in the right prefrontal cortex [Beta 114.0 (95% CI 58.4, 169.5), *q*-value 0.01, Power = 0.94]. There was no suggestion of lateralization in the other conditions, except during the 3-back in the WALK condition (Figure 2B and Supplementary Table 5), there was an indication of a decrease in Oxy-Hb predominantly in the right prefrontal cortex among those with low arterial stiffness [Beta −89.8 (95% CI −146.9, −32.8), *q*-value 0.06, Power = 0.72]. The decrease during the 3-back in the WALK condition was significantly different from baseline, but only in those with low arterial stiffness across the whole prefrontal cortex [Beta −161.1 (95% CI −235.4, −86.8), *q*-value 0.001, Power = 0.96], with a similar decrease in both the left [Beta −166.0 (95% CI −257.3, −74.6), *q*-value 0.01, Power = 0.86] and right prefrontal cortex [Beta −146.8, (95% CI −242.1, −51.5), *q*-value 0.03, Power = 0.70]. There was a significant time-by-condition interaction for the 3-back vs. baseline in those with low arterial stiffness, with no lateralization effects, such that the decrease in Oxy-Hb during the 3-back vs. baseline was significantly different from the SOCIAL condition where there was a non-significant increase in Oxy-Hb.

### Secondary Outcomes: Effects on Cognitive Performance, Psychological Factors, and Post-prandial Glucose

After the WALK condition, 3-back reaction time was faster, but no change was observed in accuracy (Table 3), and there were no significant time-by-condition interaction effects. For the psychological variables, a significant time-by-condition effect was seen between the WALK and SOCIAL conditions for positive affects. There was a significant increase in positive affects after the WALK condition compared with the SOCIAL condition. There were no significant effects of the interventions on negative affects, nor on the measure of sleepiness. There was a statistically significant increase in alertness after the WALK condition, and there was a time-by-condition interaction between the WALK and SOCIAL conditions. There were significant within condition changes in cortisol levels for all three conditions; and significant time-by-condition effects, demonstrating greater decreases in cortisol after the SOCIAL compared with the WALK and SRA conditions (Table 3). Post-prandial glucose levels (Supplementary Table 6) during the interventions did not differ between the conditions, nor were there time-by-condition interaction effects.

**TABLE 3:** Mean and standard deviation (SD) at pre- and post-test for each condition; and beta-coefficient for changes within conditions and time-by-condition interactions.

	**Conditions**						**Change within conditions (Post minus Pre)**			**Change in post-pre between conditions**		
	
	**Pre-test**			**Post-test**			**SOCIAL**	**WALK**	**SRA**	**WALK-SOCIAL**	**SRA-SOCIAL**	**WALK-SRA**
							
	**SOCIAL**	**WALK**	**SRA**	**SOCIAL**	**WALK**	**SRA**	**Beta**	**Beta**	**Beta**	**Beta**	**Beta**	**Beta**
**Cognitive function**												
1-back ACC (score)	39.0 (2.5)	39.2 (1.2)	38.7 (2.1)	39.2 (1.1)	39.2 (1.2)	39.4 (1.0)	0.2	0.1	0.7	−0.1	0.5	−0.6
1-back RT (ms)	551.9 (64.3)	567.6 (95.7)	576.7 (104.3)	562.1 (98.0)	565.1 (95.0)	584.7 (97.2)	10.2	−2.5	8.0	−12.7	−2.2	−10.5
2-back ACC (score)	75.0 (6.6)	76.9 (3.2)	75.2 (5.0)	76.8 (3.3)	76.2 (2.7)	76.0 (3.9)	1.8	−0.8	0.8	−2.6	−1.1	−1.5
2-back RT (ms)	605.5 (105.2)	594.3 (101.9)	618.8 (104.2)	611.2 (104.0)	585.4 (106.9)	624.0 (115.7)	5.6	−8.9	5.2	−14.5	−0.5	−14.0
3-back ACC (score)	68.9 (6.4)	69.3 (5.9)	68.8 (9.1)	68.2 (6.3)	68.3 (7.8)	67.8 (7.7)	−0.7	−1.0	−1.1	−0.3	−0.4	0.1
3-back RT (ms)	702.9 (98.3)	707.7 (106.8)	703.2 (120.8)	712.5 (100.1)	670.4 (118.8)	700.3 (122.0)	9.6	−37.3^*^	−2.9	−46.9	−12.5	−34.4
Positive affects (score)	31.2 (6.6)	28.1 (7.1)	29.7 (6.7)	28.0 (7.1)	31.2 (8.4)	28.8 (7.6)	−3.2	3.1	−0.8	6.2^*^	2.3	3.9
Negative affects (score)	12.0 (2.3)	12.9 (3.9)	11.4 (2.3)	11.8 (2.1)	11.6 (2.7)	11.2 (1.7)	−0.2	−1.3	−0.8	−1.1	−0.1	−1.0
KSS-sleepiness (score)	4.5 (2.1)	4.9 (1.9)	4.4 (1.9)	4.9 (1.7)	3.9 (1.8)	4.2 (1.5)	0.4	−1.0	−0.2	−1.4	−0.5	−0.8
Alertness (cm)	5.4 (2.3)	4.7 (2.6)	5.3 (2.4)	4.9 (2.4)	6.4 (2.5)	5.7 (2.6)	−0.5	1.7^**^	−0.2	2.2^*^	0.8	1.4
Stress (cortisol ng/ml)	7.2 (3.5)	4.8 (2.1)	4.6 (2.1)	3.2 (1.8)	3.2 (2.8)	2.8 (2.0)	−4.0^**^	−1.6^*^	−1.7^**^	2.5^*^	2.3^*^	0.2

*SOCIAL, social break condition; WALK, walking break condition; SRA, simple resistance activity break condition; ACC, accuracy; RT, reaction time; Positive and negative affects taken from the PANAS questionnaire; Alertness ranges from 0 “not at all” to 10 “completely alert” (highest) cm; KSS, Karolinska sleepiness questionnaire, ranges from 1 “extremely alert” to 9 “very sleepy, great effort to keep awake, fighting sleep.”*

^*^p ≤ 0.05; **p ≤ 0.001.

## Discussion

This randomized crossover study of middle-aged adults found that interrupting extended periods of sitting with frequent, short walking breaks decreased cognitive task-related right prefrontal cortex activation (as measured by Oxy-Hb) during a high workload working memory task. Corresponding improvements on some aspects of working memory performance and psychological factors were also demonstrated from the walking intervention. However, the change in working memory did not differ between the interventions. These findings suggest that frequent, short physical activity breaks during a prolonged day of sitting may to some extent administer positive effects on cognitive performance, while task-related increases in prefrontal cortex activation may not be a predominant underpinning mechanism by which this occurs. Specifically, during the high mental workload task (3-back test), decreases in right prefrontal cortex activation were seen after the walking break condition, yet with a coinciding enhancement in reaction time. However, the changes in prefrontal cortex activation after the walking condition were not significantly different from the other conditions. After stratifying by arterial stiffness, the decrease in prefrontal cortex activation during the 3-back was more pronounced in those with low arterial stiffness across the whole prefrontal cortex. This was significantly different from the prolonged sitting condition. Alertness and mood also improved after the walking breaks compared with the sitting condition. Decreases in stress were significantly greater after the sitting condition compared with both the physical activity conditions.

### Physical Activity Breaks and Prefrontal Cortex Oxygenated Hemoglobin

Decreases in prefrontal cortex activation induced by acute exercise are not unexpected and can indicate neural efficiency. Two previous studies using fNIRS reported decreases in prefrontal cortex activation, with coinciding improvements on prefrontal-related cognitive performance, although using different cognitive tasks (Murata et al., 2015; Moriarty et al., 2019). Inconsistencies in the direction of the Oxy-Hb changes used to infer cortical activation across fNIRS studies can be attributed to differences in study designs and processing procedures applied to the data. Some studies of a similar experimental design to the present one, examining physical activity breaks during prolonged sitting, have reported decreases in cerebral blood flow velocity in the middle cerebral artery compared with uninterrupted prolonged sitting (Perdomo et al., 2019; Wheeler et al., 2019). fMRI studies have also demonstrated this effect in randomized controlled trials (RCTs) of longer and shorter physical activity training among middle-aged and older adults (Maass et al., 2015; Coetsee and Terblanche, 2017; Olivo et al., 2021). A recent RCT of older adults found reduced gray matter cerebral blood flow in the frontal region with coinciding improvements on the N-back test after acute exercise compared with those who only rested (Olivo et al., 2021). Neubauer and Fink (2009) explain the theory of neural efficiency as persons having the ability to adapt to higher cognitive processing with a decreased cortical activation after the cessation of exercise. In this case, the brain is better at managing more difficult cognitive tasks with less blood flow, indicating an improved efficiency. A potential anticipatory effect may have led to enhanced cortical activation at pre-test in the WALK condition as indicated by significantly higher sedentary time the day prior, nevertheless the physical activity breaks were able to mitigate the negative circumstances resulting in an improvement on the 3-back task performance at post-test. Thus, physical activity breaks may have led to a decreased requirement for Oxy-Hb during the 3-back task. Support for the neural efficiency theory may also be evident in the results on hemispheric differences, where the decreases in Oxy-Hb were significant only in the right hemisphere. Post-exercise cortical activation normally has a left dominance. Therefore, redistribution of the blood may have occurred leading to less Oxy-Hb demand in the right hemisphere after the walking breaks in order to complete the high workload cognitive task. Young adults have been observed to have a predominate left hemisphere lateralization measured with fNIRS, after exercise during a cognitive task, whereas older adults have no lateralization effects (Yanagisawa et al., 2010; Vermeij et al., 2012; Byun et al., 2014). However, since the present study is a healthy middle-aged population a prefrontal compensatory lateralization mechanism may have transpired.

Moreover, there was a significant increase in activation during the WALK from pre-test to post-test during the 2-back, although not significantly different from the baseline. This high activation may explain the subsequent decrease in cortical activation during the 3-back as there may not have been adequate rest time between blocks to allow the hemodynamic response to go back to resting values. Additionally, the lack of randomization in the N-back test order could have increased anticipatory effects. Nevertheless, improvements in reaction time during the 3-back test were still observed signifying an effect of the walking breaks.

The moderating effect of arterial stiffness further elucidates the mechanistic effects of cerebral oxygenation. The positive effects of physical activity breaks on cognition may be more pronounced in people with better vascular health. Enhanced cardiovascular fitness is associated with reduced arterial stiffness (Goldberg et al., 2012) and can impact the structure and function of the brain (Colcombe and Kramer, 2003; Colcombe et al., 2006). Participants with superior aerobic fitness may therefore be more physiologically adept to manage the demands of the physical activity bouts and thus gain more cognitively (Pontifex et al., 2019). Interestingly, some studies have shown reduced frontal-parietal activation (measured using fMRI) during a high load working memory task, with decreases observed in the right prefrontal cortex, after 5 weeks of training in both younger and older adults compared with controls (Brehmer et al., 2014). Although this study had a longer duration of training than the current study, similar training-induced efficiency effects in brain processing could have occurred as the population in the present study was highly fit. Indeed, in an exploratory analysis we found that 3-back reaction time performance was significantly improved in those with low arterial stiffness during the WALK condition (Supplementary Figure 3).

Similarly, a previous study of young adults also showed no effects of frequent, short resistance types of breaks on cognitive function (Charlett et al., 2021). Contrarily, resistance exercises have been beneficial for glucose control and blood pressure acutely, however, in persons with diabetes (Dempsey et al., 2016a, b). SRA breaks were also found in a study of inactive office workers to acutely improve neuroplasticity, using transcranial magnetic stimulation (Bojsen-Møller et al., 2020). Thus, the lack of an effect in the present study may be due to the participants being generally healthy.

The differences in results between the previous research and our results should also be considered in light of the use of short-separation channels during the fNIRS measurement. This approach, in conjunction with advanced statistical methods, has been demonstrated to be the most accurate approach in dealing with physiological confounders (Santosa et al., 2020), but has not been used in this type of study design previously and should be considered in future studies.

### Cognitive Function

Other studies that have measured cognitive function after frequent, short physical activity breaks compared with prolonged, uninterrupted sitting, have shown beneficial effects on reaction time in young adult women (Chrismas et al., 2019), but not in overweight/obese middle-aged adults (Wennberg et al., 2016). In our study, participants improved their reaction time but not their accuracy during the 3-back test after the WALK condition. Reaction time has been suggested to be more responsive to physical activity than accuracy (McMorris et al., 2011), due to acute increases in catecholamines (e.g., norepinephrine) after exercise (McMorris et al., 2011; da Silva de Vargas et al., 2017).

Conflicting results with previous research findings may also be due to the moderating effects of timing, duration, and intensity of the physical activity, as well as the timing and type of cognitive test employed after the intervention (Chang et al., 2012). While our study investigated cognitive effects of different types of breaks, further investigations are needed to elucidate how durable the effects are.

Furthermore, employing the N-back test in the current study additionally allowed for testing different mental workloads. No effect was observed in the 2-back test in the present study, which may result from the test not being sufficiently difficult. Indeed discrimination is best when the degree of difficulty is most distinct, such as from the high demanding 3-back test, as seen in a study by Herff et al. (2014) of young adults, and in the present study. However, there have been minimal investigations previously performed looking into the effects of short bouts of exercise on various cognitive workloads. One study in older adults found no effect of acute exercise on either the 1-, 2-, or 3-back tests (Olivo et al., 2021), although a decrease in performance was exhibited with increasing load. There was, nonetheless, an indication of improvement only on the 3-back test for the exercise group compared with the resting group (Olivo et al., 2021). Furthermore, another study of older adults saw improvements on the 2-back test 15-min post exercise cessation (Stute et al., 2020). It is suggested that low intensity exercise may produce immediate positive effects on cognition, whereas higher intensities may have a delayed effect (Chang et al., 2012). Although there is a general idea that exercise produces beneficial effects on cognitive performance, this has mainly been observed in persons with cognitive impairment and dependent on the type of cognitive test measured (Chang et al., 2012). Immediate improvements have been observed from a 10-min bout of moderate exercise on executive function tests of inhibition, such as the Stroop test, in both younger and older adults, with also increases in Oxy-Hb (Yanagisawa et al., 2010; Hyodo et al., 2012). However, the physiological differences from increasing task demands requires further study. It is believed that cognitive load may affect both behavioral and hemodynamic responses differently in younger and older adults, and high fitness may compensate for declines from greater task demand and age (Agbangla et al., 2019). This may be the case in the current study’s middle-aged population, where effects from the walking breaks were observed only among those with low arterial stiffness. Thus, not only timing, duration, and intensity of the physical activity need to be taken into consideration, but also age of the population, and the load and type of the cognitive task in relation to behavioral and hemodynamic responses.

### Psychological Outcomes

Acute exercise has previously been found to elicit positive effects on perceived attention and mood, compared with those who were sedentary for 2 h (Niedermeier et al., 2020). Even though low intensity physical activity breaks and even less intense shoulder and neck exercise breaks positively impacted subjective sleepiness in sleep restricted adults (Sallinen et al., 2008; Vincent et al., 2018), we did not see any effect of the physical activity breaks on sleepiness. In fact, our participants had adequate sleep prior to test days (see Table 2).

Decreases in cortisol levels, denoting stress, was observed after all conditions, but with the greatest changes after the SOCIAL condition. Physical activity breaks may incite a stress reaction that, at moderate levels, elevates attention. Another study found no difference in salivary cortisol levels after sitting uninterrupted for 180 min compared with sitting with physical activity breaks (Sperlich et al., 2018). Salivary cortisol can indicate circulating cortisol from training stress and psycho-physiological stress reactions to exercise, activating the hypothalamic-pituitary-adrenal axis (Wang et al., 2019). Therefore, it is not surprising that the reduction in cortisol is largest after sitting. Post-prandial glucose levels were not significantly different between the conditions, as similarly observed in previous studies (Hansen et al., 2016; Sperlich et al., 2018). This was expected as the participants were relatively healthy.

### Other Potential Mechanisms

Increases in catecholamines (e.g., norepinephrine) (McMorris et al., 2011; da Silva de Vargas et al., 2017) or neurotrophic factors may have also influenced the results, although not measured in the present study. Neurotrophic factors are known to increase after acute bouts of exercise and positively impact cognitive function (Nilsson et al., 2020). Specifically, increases in serum brain-derived neurotrophic factor has been observed after conditions of interrupted sitting with frequent, short physical activity breaks compared with uninterrupted sitting, in conjunction with improvements in working memory (Wheeler et al., 2020). In addition, increases in lactate may also explain the improvements in cognitive performance from acute exercise. One study showed that after both a bout of moderate-intensity exercise and high intensity interval exercise there were increases in blood lactate levels, which the authors explained as a potential reason for the improvements on a test of executive function (Tsukamoto et al., 2016). Thus, lactate may replace glucose as the main energy source after acute exercise in order to help in the maintenance of cognitive performance.

### Strengths and Limitations

Strengths of our study include registration of our trial and publication of the protocol to add rigor to the analyses and findings. In addition, the use of short-separation channels in combination with a robust statistical approach ideally dealt with systemic contamination and motion artifacts, which augmented the accuracy of the results. Limitations include that mind wandering or other sources of arousal could have occurred during recovery periods of the N-back test, which may have induced changes in Oxy-Hb. This may also be a result of inadequate resting periods between blocks of the N-back, prohibiting the hemodynamic response to go back to baseline levels. Additionally, a lack of pseudo-randomization of N-back tests may have induced anticipatory effects (Yücel et al., 2021). Future experimental designs should take this into consideration. Repositioning of the cap during test days may have also led to slight misplacement of the optodes from pretest to posttest; however, the use of a marker on the forehead helped in maintaining accuracy in cap placement as best as possible. Since we only investigated the prefrontal cortex, activity-related changes in other regions cannot be excluded. Our study was powered to detect between-condition differences in Oxy-Hb, but was less sensitive to changes in cognitive performance. Consequently, more investigations are necessary to assess cognitive effects. Even though the power calculation generated the sample size used in this study, the calculation was based off studies with slightly different experimental designs, thus more studies should be employed with a similar approach to confirm the results and if larger sample sizes are needed. The participants in our study were relatively fit and healthy. Therefore, our findings might not be applicable to populations that are more vulnerable. The finding that arterial stiffness moderates the cerebrovascular response, stresses the need for future investigations to specifically target individuals with high arterial stiffness. Finally, although the inclusion of a control group may be considered more advantageous in order to eliminate bias, the crossover design with randomly ordered, yet counter balanced sessions, alternatively, permitted participants to serve as their own controls and thus, hold constant any variation between individuals.

## Conclusion

Interrupting prolonged sitting, with frequent, short walking breaks decreased task-related right prefrontal cortex activation as measured by Oxy-Hb during a high load working memory task. Still, frequent, short walking breaks also enhanced working memory performance, suggesting that physical activity breaks during prolonged sitting may help preserve or even improve neural efficiency. Of further importance, alertness and positive mood were enhanced by frequent, short walking breaks compared with prolonged sitting. While more experimental scrutiny is needed to clarify the physiological mechanisms underlying such improved neural efficiency, frequent, short walking breaks may be recommended in middle-aged adults to support psychological well-being during extended periods of sitting and cognitive performance on mentally demanding tasks.

## Data Availability Statement

The datasets presented in this article are not readily available because of ethical restrictions. Requests to access the datasets should be directed to MK, maria.ekblom@gih.se.

## Ethics Statement

The studies involving human participants were reviewed and approved by the Swedish Ethical Review Authority, Stockholm, Sweden (Dnr 2019-00998). The patients/participants provided their written informed consent to participate in this study.

## Author Contributions

EH, ÖE, OT, MF, CE, and ME: conceptualization, methodology, and writing – review and editing. EH, ÖE, OT, MF, and ME: data curation and investigation. EH, ÖE, and OT: formal analysis. ME and ÖE: funding acquisition and project administration. ÖE, CE, and ME: supervision. EH, OT, and ME: validation. EH, OT, and CE: visualization. EH: roles/writing – original draft. All authors contributed to the article and approved the submitted version.

## Conflict of Interest

The authors declare that the research was conducted in the absence of any commercial or financial relationships that could be construed as a potential conflict of interest.

## Publisher’s Note

All claims expressed in this article are solely those of the authors and do not necessarily represent those of their affiliated organizations, or those of the publisher, the editors and the reviewers. Any product that may be evaluated in this article, or claim that may be made by its manufacturer, is not guaranteed or endorsed by the publisher.

## Footnotes

1
https://github.com/huppertt/nirs-toolbox
