# Supplementary material for: Frequent, Short Physical Activity Breaks Reduce Prefrontal Cortex Activation but Preserve Working Memory in Middle-Aged Adults: ABBaH Study
Source: Front Hum Neurosci. 2021 Sep 16;15:719509. doi: 10.3389/fnhum.2021.719509 (PMC8481573; doi:10.3389/fnhum.2021.719509)
Supplement: Supplementary file 1 [file Data_Sheet_1.docx]

Supplementary Material

1. **Supplementary material – Fitness testing and ascertainment of speed/inclination of the treadmill for the WALK break condition (Heiland EG et al. 2020. *Front. Hum. Neurosci.*)**

In order to assess *fitness* first a general health screening was performed, where contraindications for the fitness test were checked, and body mass (in kilograms) and height (in centimetres) were measured. The participant was fitted with a heart rate monitor (Polar Oy, Kempere, Finland). Heart rate during the end of the two walking speeds and the end of the maximal treadmill test was saved.

In a first test, the participant walked at two different speeds and/or inclinations for 4 min each, with one being an easy walk and one a very brisk walk. The heart rate from these two work rates was used to interpolate or extrapolate the treadmill speed and inclination needed to elicit 75-80% of maximal heart rate. This intensity was later used as work intensity in the WALK experimental arm.

After the walking test, a 5 min jogging warm up was performed on the same treadmill, starting at a self-selected speed and inclination. During the warm-up the subject practiced grabbing the treadmill handlebars and exiting the treadmill safely, so that they felt comfortable and acquainted with the stopping technique. At the end of the warm-up, inclination typically was 1 degree and the speed between 8-10 km per hour.

After the warm-up, the participant was fitted with a face-mask that was connected to a computerized metabolic system (OxyCon Pro, Erich Jaeger GmbH, Hochenberg, Germany). The system was calibrated before each measurement. The inspiratory flow meter was checked with the low and high airflow procedure. The gas analyzer calibration was performed using a gas mixture of 15.0% oxygen and 6.0% carbon dioxide (AirLiquide, Paris, France). During data collection, the breath-by-breath mode was used.

The maximal treadmill test started at a 1 degree inclination and the same speed as at the end of the warm-up. The maximal test protocols were individually designed and executed and the speed and/or inclination were increased every minute until volitional exhaustion. The participants were given verbal encouragement during the test.

Oxygen consumption (VO_2_) data were averaged over 10 epochs. Criterion for achieving VO_2_ peak was a levelling off in oxygen consumption despite an increase in elevation and/or speed. The mean of the three highest 10 s periods were saved as the peak VO_2_ expressed both as absolute terms (L oxygen consumed per minute, L x min^-1^) and relative terms (mL oxygen consumed per minute and kilograms of body mass, mL x min^-1^ x kg^-1^).

# Supplementary Figure 1





**Supplementary Figure 1.** Prefrontal cortex montage setup for the fNIRS cap with 8 LED light sources (S1-S8) and 7 detectors (D1-D7) placed according to the standard 10-20 system, with a source-detector separation of 3 cm. An eighth detector was split into eight short-separation detectors (SD1-SD8) placed at a distance of 0.8 cm from each source, to capture superficial blood flow.

#
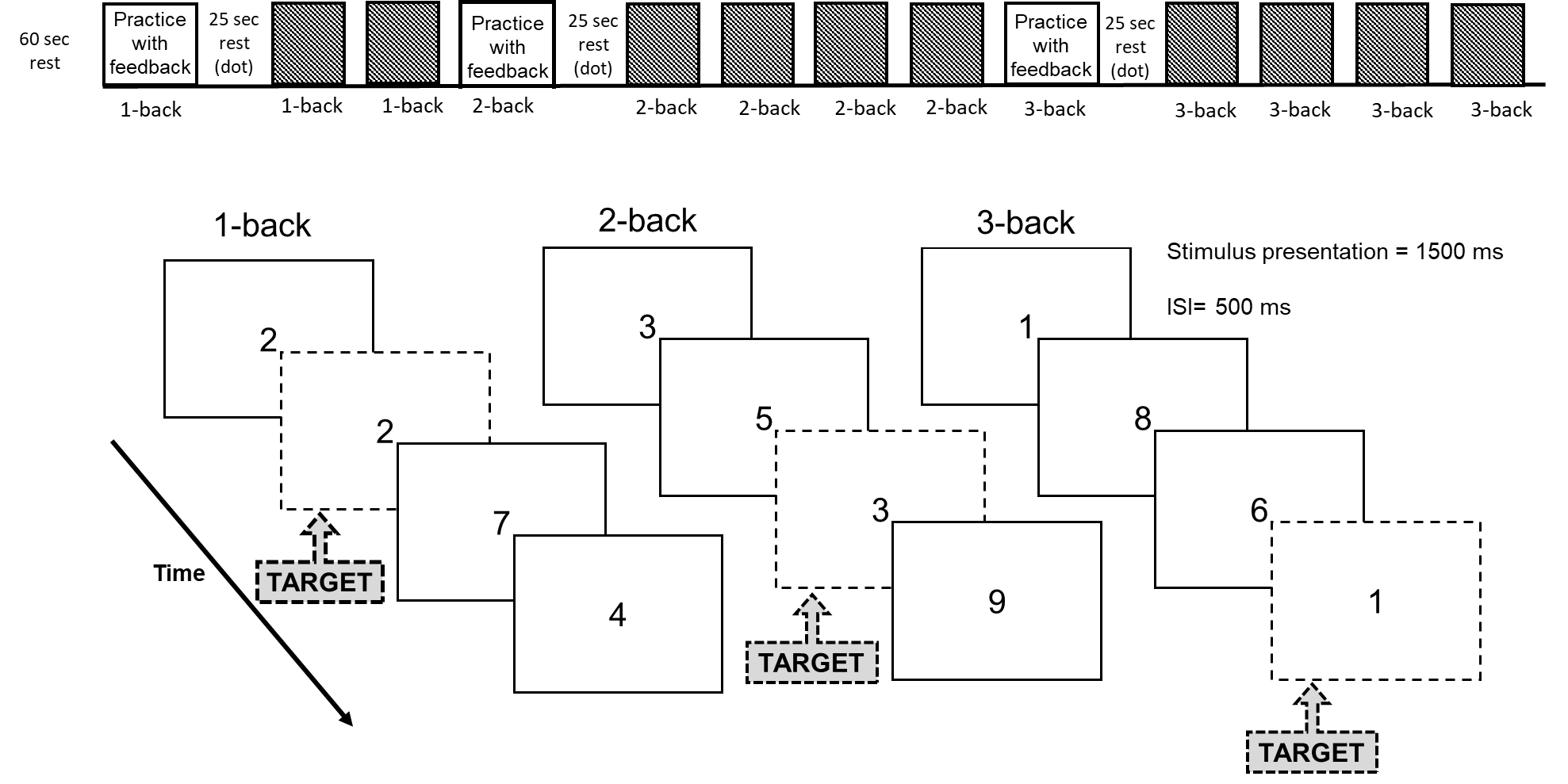

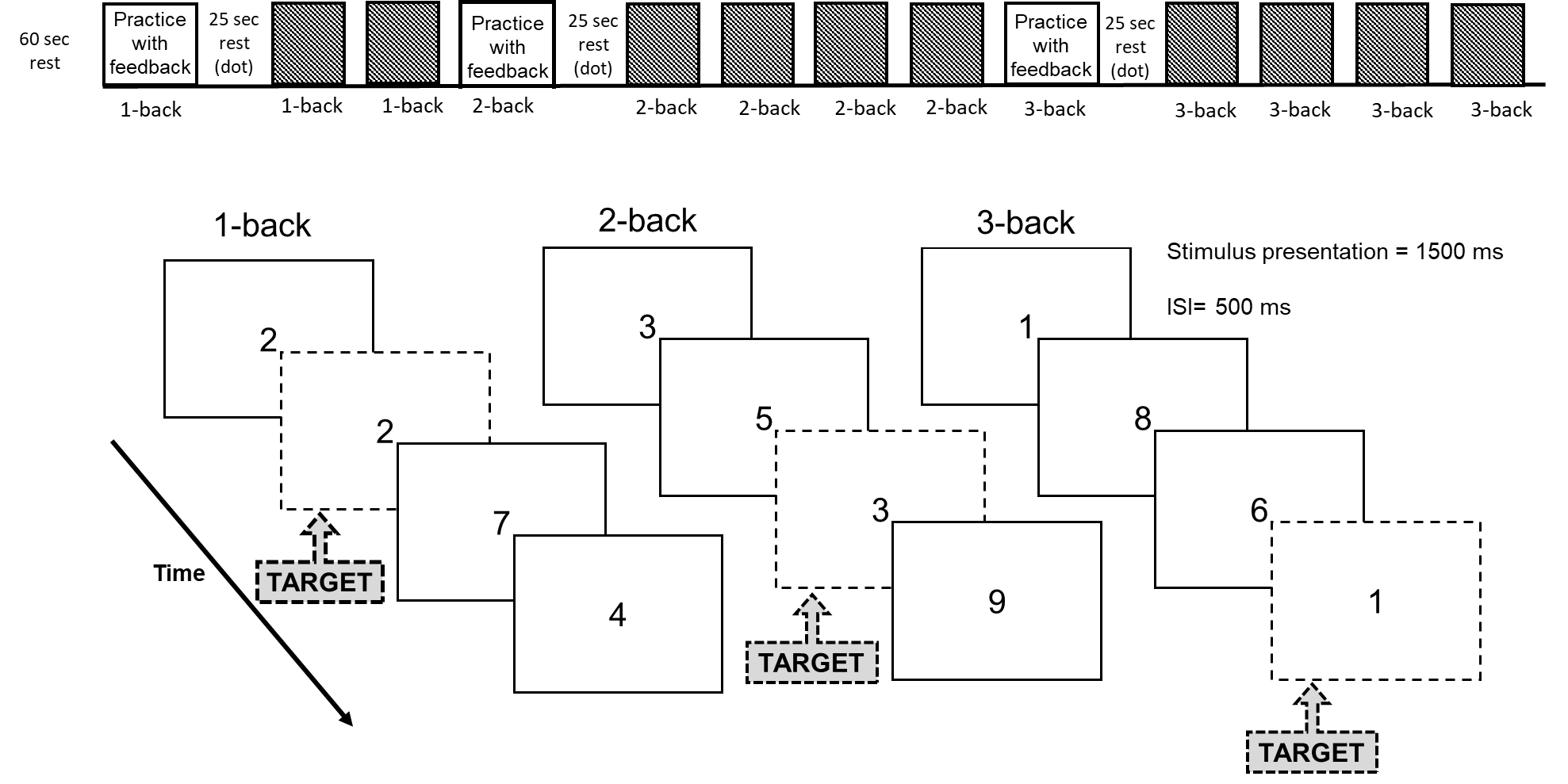
Supplementary Figure 2

**Supplementary Figure 2.** Schematic of computer-based, numerical working memory cognitive tests administered simultaneously with functional near-infrared spectroscopy. Initially, each participant was required to stare at a white dot on a black screen for 60 secs. Prior to each N-back test without feedback, a 40-sec practice session with feedback was performed, followed by 25 secs of rest involving staring at a dot on the screen. The 1-back entailed two blocks of 20 digits (used as baseline), and four blocks of 20 digits for 2- and 3-back tests. Participants were required to answer with a key press within 2 secs of onset whether the stimulus was correct or not. Each stimulus was presented for 1500 ms with an interstimulus interval (ISI) of 500 ms. Instructions and a reminder of which test was next was presented before each block, followed by a prompt to press the spacebar to continue. An example of a correct response is labeled as “Target”.

# Supplementary Tables

| **Supplementary Table 1.** Within condition, pre-test to post-test changes in oxygenated- and deoxygenated-hemoglobin in the whole, left, and right prefrontal cortex (PFC) during 2-back and 3-back cognitive tests (vs. 1-back). | | | | | |
| --- | --- | --- | --- | --- | --- |
|  |  |  | **2-back vs 1-back** | | |
|  |  |  | Beta (95% confidence interval) | | |
| Condition | Time | Parameter | WHOLE PFC | LEFT PFC | RIGHT PFC |
| SOCIAL | POST-PRE | Oxy-Hb | -50.8 (-104.7, 3.2) | -85.6 (-153.1, -18.1) | -2.9 (-71.9, 66.1) |
|  |  | d-Oxy-Hb | 7.9 (-19.7, 35.5) | 1.9 (-33.0, 36.8) | 13.3 (-22.8, 49.4) |
|  |  |  |  |  |  |
| WALK | POST-PRE | Oxy-Hb | 32.5 (-10.7, 75.8) | 11.0 (-43.7, 65.7) | 60.4 (5.6, 115.1) |
|  |  | d-Oxy-Hb | 18.7 (-3.2, 40.6) | -24.8 (-52.8, 3.2) | **45.3 (17.2, 73.4)*** |
|  |  |  |  |  |  |
| SRA | POST-PRE | Oxy-Hb | -51.5 (-103.5, 0.5) | -45.3 (-109.7, 19.0) | -62.3 (-129.4, 4.9) |
|  |  | d-Oxy-Hb | -7.9 (-34.6, 18.9) | -15.9 (-49.1, 17.3) | 0.6 (-35.3, 36.5) |
|  |  |  | **3-back vs 1-back** | | |
|  |  |  | Beta (95% confidence interval) | | |
|  |  |  | WHOLE PFC | LEFT PFC | RIGHT PFC |
| SOCIAL | POST-PRE | Oxy-Hb | -12.6 (-67.4, 42.1) | -34.3 (-103.1, 34.5) | 15.3 (-54.3, 84.8) |
|  |  | d-Oxy-Hb | -2.2 (-29.9, 25.6) | -14.2 (-49.3, 20.9) | 6.2 (-30.0, 42.4) |
|  |  |  |  |  |  |
| WALK | POST-PRE | Oxy-Hb | **-76.1 (-120.2, -32.0)*** | -45.2 (-100.8, 10.5) | -70.6 (-126.5, -14.8) |
|  |  | d-Oxy-Hb | 9.9 (-12.3, 32.1) | -32.6 (-60.9, -4.3) | 40.4 (11.8, 69.0) |
|  |  |  |  |  |  |
| SRA | POST-PRE | Oxy-Hb | -37.1 (-89.5, 15.4) | -56.4 (-121.5, 8.6) | -22.6 (-90.2, 45.0) |
|  |  | d-Oxy-Hb | -18.1 (-45.0, 8.8) | -25.0 (-58.3, 8.4) | -6.3 (-42.3, 29.8) |
| Oxy-Hb, oxygenated hemoglobin; d-Oxy-­Hb, deoxygenated hemoglobin; CI, confidence interval; SOCIAL, social break condition; WALK, walking break condition; SRA, simple resistance activity break condition.**q*-value ≤0.05 (FDR-adjusted p-values for multiple comparisons). | | | | | |

| **Supplementary Table 2.** Changes in oxygenated- and deoxygenated-hemoglobin in the left and right prefrontal cortex during 1-back, 2-back, and 3-back cognitive tests, and within condition changes from pre-test to post-test. | | | | | | | | |
| --- | --- | --- | --- | --- | --- | --- | --- | --- |
|  |  |  | Beta (95% confidence interval) | | | | | |
|  |  |  | **1-back** | | **2-back** | | **3-back** | |
| Condition | Time | Parameter | LEFT | RIGHT | LEFT | RIGHT | LEFT | RIGHT |
| SOCIAL | PRE | Oxy-Hb | -58.2 (-98.0,-18.3) | 2.8 (40.9, -35.4) | -26.2 (-54.8, 2.3) | -34.4 (-7.0, -61.7) | -55.2 (-84.5, -25.9) | -34.4 (-6.7, -62.1) |
|  |  | d-Oxy-Hb | 7.7 (-14.9, 30.2) | 6.1 (-17.1, 29.3) | 18.4 (2.3, 34.6) | 19.1 (2.5, 35.8) | 6.1 (-10.2, 22.4) | 4.6 (-12.2, 21.3) |
|  | POST | Oxy-Hb | 13.5 (-26.4, 53.5) | 19.5 (62.3, -23.3) | -40.1 (-68.6, -11.6) | -20.5 (9.8, -50.8) | -17.8 (-47.1, 11.4) | -2.4 (28.5, -33.3) |
|  |  | d-Oxy-Hb | -6.7 (-25.6, 12.2) | -23.2 (-42.7, -3.7) | 6.0 (-7.8, 19.7) | 3.1 (-11.0, 17.3) | -22.5 (-36.5, -8.5) | -18.6 (-32.9, -4.3) |
|  |  |  |  |  |  |  |  |  |
| WALK | PRE | Oxy-Hb | -81.5 (-107.0, -56.0) | -23.2 (0.4, -46.7) | -55.9 (-75.3, -36.6) | -61.0 (-42.8, -79.2) | -8.5 (-29.0, 12.0) | 39.6 (59.1, 20.1) |
|  |  | d-Oxy-Hb | 1.4 (-11.3, 14.0) | 27.5 (14.5, 40.4) | 7.5 (-2.4, 17.5) | -20.2 (-30.5, -9.9) | -14.0 (-24.2, -3.7) | -16.8 (-27.6, -6.0) |
|  | POST | Oxy-Hb | -69.1 (-107.4, -30.7) | -27.7 (12.0, -67.4) | -32.5 (-60.0, -5.0) | -5.2 (23.2, -33.5) | -41.2 (-68.9, -13.5) | -35.6 (-6.9, -64.2) |
|  |  | d-Oxy-Hb | 27.7 (7.1, 48.2) | 1.2 (-19.4, 21.8) | 9.0 (-5.9, 23.9) | -1.2 (-16.0, 13.7) | -20.3 (-35.3, -5.2) | -2.6 (-17.7, 12.4) |
|  |  |  |  |  |  |  |  |  |
| SRA | PRE | Oxy-Hb | -41.7 (-80.0, -3.5) | -21.0 (17.6, -59.6) | -32.5 (-59.8, -5.1) | -8.4 (19.1, -36.0) | -10.5 (-38.4, 17.4) | -27.0 (0.8, -54.9) |
|  |  | d-Oxy-Hb | -2.2 (-22.3, 17.8) | 1.2 (-21.0, 23.4) | 3.5 (-11.0, 18.0) | -5.3 (-21.1, 10.6) | -3.1 (-17.7, 11.5) | -15.8 (-31.8, 0.2) |
|  | POST | Oxy-Hb | 6.8 (-30.5, 44.1) | 3.6 (43.7, -36.5) | -29.2 (-56.1, -2.4) | -46.1 (-17.4, -74.8) | -18.4 (-45.9, 9.2) | -25.0 (4.2, -54.3) |
|  |  | d-Oxy-Hb | 7.9 (-11.6, 27.5) | 4.0 ( -16.5, 24.5) | -2.2 (-16.4, 12.0) | -1.9 (-16.8, 12.9) | -17.9 (-32.2, -3.5) | -19.3 (-34.3, -4.3) |
| **WITHIN CONDITION POST-PRE CHANGES** | | | | | | | | |
|  |  |  | Beta (95% confidence interval) | | | | | |
|  |  |  | **1-back** | | **2-back** | | **3-back** | |
| Condition | Time | Parameter | LEFT | RIGHT | LEFT | RIGHT | LEFT | RIGHT |
| SOCIAL | POST-PRE | Oxy-Hb | 71.7 (15.7, 127.8) | 16.8 (-40.2, 73.7) | -13.9 (-53.7, 25.9) | 13.9 (-26.4, 54.1) | 37.4 (-3.4, 78.3) | 32.0 (-8.9, 73.0) |
|  |  | d-Oxy-Hb | -14.4 (-43.1, 14.3) | -29.3 (-59.0, 0.4) | -12.5 (-32.7, 7.8) | -16.0 (-36.9, 4.9) | -28.6 (-49.1, -8.1) | -23.1 (-44.2, -2.0) |
|  |  |  |  |  |  |  |  |  |
| WALK | POST-PRE | Oxy-Hb | 12.5 (-33.2, 58.1) | -4.5 (-50.2, 41.1) | 23.5 (-9.7, 56.6) | **55.8 (22.8, 88.8)*** | -32.7 (-66.7, 1.2) | **-75.2 (-109.1, -41.2)*** |
|  |  | d-Oxy-Hb | 26.3 (2.9, 49.7) | -26.3 (-49.7, -2.9) | 1.5 (-15.3, 18.3) | 19.0 (2.2, 35.8) | -6.3 (-23.4, 10.8) | 14.1 (-3.2, 31.5) |
|  |  |  |  |  |  |  |  |  |
| SRA | POST-PRE | Oxy-Hb | 48.6 (-4.5, 101.6) | 24.6 (-30.7, 79.9) | 3.2 (-34.5, 41.0) | -37.7 (-76.9, 1.6) | -7.9 (-46.5, 30.8) | 2.0 (-37.9, 41.9) |
|  |  | d-Oxy-Hb | 10.2 (-17.1, 37.5) | 2.8 (-26.8, 32.3) | -5.7 (-25.0, 13.6) | 3.3 (-17.5, 24.2) | -14.8 (-34.3, 4.7) | -3.5 (-24.6, 17.5) |
| Oxy-Hb, oxygenated hemoglobin; d-Oxy-­Hb, deoxygenated hemoglobin; CI, confidence interval; SOCIAL, social break condition; WALK, walking break condition; SRA, simple resistance activity break condition.**q*-value ≤0.05 (FDR-adjusted p-values for multiple comparisons) for within condition post-pre changes. | | | | | | | | |

| **Supplementary Table 3.**  Arterial stiffness stratified changes in oxygenated- and deoxygenated-hemoglobin in the whole prefrontal cortex and in the left and right hemispheres, during the **1-back** cognitive test, and within condition (SOCIAL, WALK, SRA) changes from pre-test to post-test. | | | | | | | |
| --- | --- | --- | --- | --- | --- | --- | --- |
|  |  | **1-back**  Beta (95% confidence interval) | | | | | |
|  |  | WHOLE PFC | | High arterial stiffness | | Low arterial stiffness | |
| **Condition**/ Time | Parameter | High arterial stiffness | Low arterial stiffness | Left PFC | Right PFC | Left PFC | Right PFC |
| **SOCIAL** |  |  |  |  |  |  |  |
| PRE | Oxy-Hb | 52.7 (13.0, 92.4) | -13.8 (-66.3, 38.6) | 51.7  (4.1, 99.2) | 38.4  (-13.5, 90.3) | -43.0  (-104.4, 18.5) | 15.5  (-54.5, 85.6) |
|  | d-Oxy-Hb | -30.9 (-50.1, -11.8) | -2.0 (-23.6, 19.6) | -14.2  (-39.1, 10.7) | -45.9  (-70.5, -21.2) | -4.6  (-30.4, 21.2) | 4.0  (-24.8, 32.9) |
| POST | Oxy-Hb | -19.0 (-59.3, 21.3) | -29.0 (-70.8, 12.7) | -36.4  ( -88.5, 15.7) | -9.1  (-60.3, 42.1) | -57.3  (-111.2,-3.3) | 3.0  (-49.1, 55.0) |
|  | d-Oxy-Hb | 10.6 (-12.2, 33.5) | 4.2 (-20.5, 28.9) | 14.6  (-15.2, 44.4) | 7.7  (-22.2, 37.7) | 2.8  (-27.8, 33.5) | 5.9  (-27.1, 38.9) |
| **WALK** |  |  |  |  |  |  |  |
| PRE | Oxy-Hb | -50.0 (-84.2, -15.8) | -9.6 (-63.0, 43.8) | -72.9  (-115.7, -30.1) | -41.5  (-85.6, 2.6) | -29.2  (-95.9, 37.6) | 15.0  (-53.5, 83.4) |
|  | d-Oxy-Hb | 14.2 (-4.2, 32.6) | 19.5 (-6.3, 45.4) | 27.3  (2.4, 52.3) | 3.7  (-20.0, 27.4) | 32.8  (3.0, 62.5) | 3.6  (-32.2, 39.5) |
| POST | Oxy-Hb | -44.4 (-70.1, -18.7) | -88.7 (-118.7, -58.7) | -49.4  (-84.6, -14.2) | -29.5  (-59.1, 0.1) | -44.2  (-179.2, -109.2) | -42.0  (-80.9, -3.1) |
|  | d-Oxy-Hb | 9.5 (-5.6, 24.7) | 38.6 (24.7, 52.6) | -16.7  (-36.6,3.3) | 23.4  (5.7, 41.1) | 23.4  (7.5, 39.4) | 45.9  (27.0, 64.8) |
| **SRA** |  |  |  |  |  |  |  |
| PRE | Oxy-Hb | 5.3 (-31.3, 41.9) | 13.4 (-35.1, 61.8) | -2.8  (-48.2, 42.6) | 9.5  (-37.2, 56.2) | 24.9  (-32.7, 82.6) | 0.9  (-63.3, 65.2) |
|  | d-Oxy-Hb | 1.7 (-17.3, 20.7) | 12.8 (-9.2, 34.8) | 3.3  (-21.2, 27.8) | -0.3  (-25.1, 24.5) | 12.8  (-13.9, 39.5) | 9.5  (-20.4, 39.5) |
| POST | Oxy-Hb | -49.1 (-87.8, -10.5) | -4.6 (-46.4, 37.2) | -65.1  (-114.8, -15.4) | -33.0  (-81.3, 15.2) | -13.8  (-65.0, 37.4) | -6.3  (-60.7, 48.0) |
|  | d-Oxy-Hb | 19.1 (-3.4, 41.6) | -16.2 (-36.8, 4.4) | 5.7  (-22.4, 33.9) | 30.5  (0.2, 60.8) | -8.2  (-33.3, 16.9) | -22.9  (-51.0, 5.1) |
| **WITHIN CONDITION POST-PRE CHANGES** | | | | | | | |
|  |  | **1-back**  Beta (95% confidence interval) | | | | | |
|  |  | WHOLE PFC | | High arterial stiffness | | Low arterial stiffness | |
| **Condition**/ Time | Parameter | High arterial stiffness | Low arterial stiffness | Left PFC | Right PFC | Left PFC | Right PFC |
| **SOCIAL** |  |  |  |  |  |  |  |
| POST-PRE | Oxy-Hb | **71.7 (15.4, 127.9)*** | 15.2 (-51.6, 81.9) | 88.1  (18.0, 158.2) | 47.5  (-24.9, 120.0) | 14.3 (-67.0, 95.6) | 12.6  (-74.3, 99.4) |
|  | d-Oxy-Hb | **-41.6 (-70.8, -12.3)*** | -6.2 (-38.4, 26.0) | -28.8  (-66.9, 9.3) | -53.6  (-91.6, -15.6) | -7.5 (-46.6, 31.7) | -1.9  (-44.9, 41.1) |
| **WALK** |  |  |  |  |  |  |  |
| POST-PRE | Oxy-Hb | -5.6 (-48.0, 36.8) | **79.1 (18.2, 140.0)*** | -23.5  (-78.4, 31.5) | -12.0  (-64.5, 40.4) | 115.0 (40.0, 190.1) | 57.0  (-21.2, 135.2) |
|  | d-Oxy-Hb | 4.6 (-18.5, 27.8) | -19.1 (-47.8, 9.5) | 44.0  (12.9, 75.1) | -19.7  (-48.2, 8.8) | 9.3 (-23.4, 42.0) | -42.2  (-81.8, -2.7) |
| **SRA** |  |  |  |  |  |  |  |
| POST-PRE | Oxy-Hb | 54.4 (1.5, 107.4) | 18.0 (-45.6, 81.6) | 62.3  (-4.5, 129.2) | 42.5  (-24.2, 109.3) | 38.8 (-37.9, 115.4) | 7.3  (-76.4, 91.0) |
|  | d-Oxy-Hb | -17.4 (-46.4, 11.5) | 29.0 (-0.5, 58.5) | -2.4  (-39.0, 34.1) | -30.8  (-69.3, 7.6) | 21.1 (-14.6, 56.7) | 32.5  (-7.6, 72.6) |
| Oxy-Hb, oxygenated hemoglobin; d-Oxy-­Hb, deoxygenated hemoglobin; CI, confidence interval; SOCIAL, social break condition; WALK, walking break condition; SRA, simple resistance activity break condition; PFC, prefrontal cortex.**q*-value ≤0.05 (FDR-adjusted p-values for multiple comparisons) for within condition post-pre changes. | | | | | | | |

| **Supplementary Table 4.**  Arterial stiffness stratified changes in oxygenated- and deoxygenated-hemoglobin in the whole prefrontal cortex and in the left and right hemispheres, during the **2-back** cognitive test, and within condition (SOCIAL, WALK, SRA) changes from pre-test to post-test. | | | | | | | |
| --- | --- | --- | --- | --- | --- | --- | --- |
|  |  | **2-back**  Beta (95% confidence interval) | | | | | |
| **Condition**/ Time | Parameter | WHOLE PFC | | High arterial stiffness | | Low arterial stiffness | |
|  |  | High arterial stiffness | Low arterial stiffness | Left PFC | Right PFC | Left PFC | Right PFC |
| **SOCIAL** |  |  |  |  | |  | |
| PRE | Oxy-Hb | -14.5 (-42.5, 13.4) | -39.8 (-77.4, -2.2) | -14.9  (-48.6, 18.9) | -15.6  (-52.0, 20.8) | -75.9  (-120.1, -31.7) | -7.8  (-57.7, 42.2) |
|  | d-Oxy-Hb | -3.7 (-17.5, 10.0) | 14.6 (-1.2, 30.3) | -1.2  (-19.1, 16.7) | -2.1  (-19.8, 15.5) | 15.4  (-3.6, 34.4) | 10.3  (-10.6, 31.3) |
| POST | Oxy-Hb | -58.6 (-87.4, -29.9) | 3.5 (-26.5, 33.5) | -64.6  (-101.7, -27.5) | -58.6  (-95.1, -22.0) | 7.6  (-31.3, 46.5) | -11.1  (-48.4, 26.3) |
|  | d-Oxy-Hb | 19.1 (2.8, 35.4) | 18.6 (0.9, 36.4) | 15.9  (-5.5, 37.2) | 24.4  (3.0, 45.8) | 22.3  (0.3, 44.4) | 12.4  (-11.3, 36.2) |
| **WALK** |  |  |  |  |  |  |  |
| PRE | Oxy-Hb | -12.6 (-37.2, 11.9) | -5.7 (-43.4, 32.1) | -41.2  (-71.9, -10.5) | 9.8  (-21.9, 41.5) | -1.4  (-48.9, 46.1) | -20.8  (-68.9, 27.3) |
|  | d-Oxy-Hb | 1.8 (-11.6, 15.1) | 5.3 (-13.0, 23.7) | 7.7  (-10.4, 25.8) | -1.6  (-18.7, 15.6) | 11.5  (-9.8, 32.8) | 0.3  (-25.1, 25.7) |
| POST | Oxy-Hb | -54.8 (-74.3, -35.3) | -97.7 (-120.1, -75.3) | -83.8  (-110.5, -57.1) | -20.2  (-42.6, 2.2) | -53.3  (-79.4,-27.3) | -134.8  (-163.9, -105.7) |
|  | d-Oxy-Hb | 2.7 (-8.7, 14.1) | -26.6 (-37.4, -15.9) | 25.5  (10.4, 40.5) | -16.1  (-29.8, -2.5) | -10.1  (-22.7, 2.5) | -36.9  (-51.5, -22.4) |
| **SRA** |  |  |  |  |  |  |  |
| PRE | Oxy-Hb | -6.8 (-33.0, 19.5) | -83.9 (-118.4, -49.4) | -8.9  (-41.6, 23.8) | -12.1  (-45.5, 21.3) | -64.2  (-105.3, -23.1) | -102.8  (-148.5, -57.0) |
|  | d-Oxy-Hb | -6.9 (-20.7, 6.9) | -1.8 (-17.7, 14.0) | -5.4  (-23.3, 12.6) | -6.4  (-24.3, 11.6) | -2.5  (-21.8, 16.8) | -1.7  (-23.3, 19.8) |
| POST | Oxy-Hb | -15.2 (-42.8, 12.4) | -26.5 (-56.2, 3.2) | -30.9  (-66.4, 4.6) | -4.2  (-38.7, 30.3) | -36.5  (-72.9, -0.1) | -17.6  (-56.3, 21.0) |
|  | d-Oxy-Hb | 4.6 (-11.5, 20.7) | -4.3 (-19.0, 10.5) | 10.7  (-9.6, 31.0) | 2.6  (-19.1, 24.3) | -0.3  (-18.3, 17.8) | -8.9  (-29.0, 11.1) |
| **WITHIN CONDITION POST-PRE CHANGES** | | | | | | | |
|  |  | **2-back**  Beta (95% confidence interval) | | | | | |
|  |  | WHOLE PFC | | High arterial stiffness | | Low arterial stiffness | |
| **Condition**/ Time | Parameter | High arterial stiffness | Low arterial stiffness | Left PFC | Right PFC | Left PFC | Right PFC |
| **SOCIAL** |  |  |  |  |  |  |  |
| POST-PRE | Oxy-Hb | 44.1 (4.5, 83.7) | -43.3 (-91.0, 4.4) | 49.7  (0.2, 99.3) | 42.9  (-8.0, 93.8) | -83.5  (-141.7, -25.2) | 3.3  (-58.5, 65.1) |
|  | d-Oxy-Hb | -22.9 (-43.4, -2.3) | -4.0 (-26.9, 18.8) | -17.1  (-43.8, 9.7) | -26.6  (-53.2, 0.1) | -6.9  (-34.8, 20.9) | -2.1  (-32.6, 28.4) |
| **WALK** |  |  |  |  |  |  |  |
| POST-PRE | Oxy-Hb | **42.2 (11.4, 73.0)*** | **92.0 (48.6, 135.5)*** | 42.6  (2.5, 82.7) | 30.0  (-7.9, 67.9) | 52.0  (-1.7, 105.6) | **114.0**  **(58.4, 169.5)*** |
|  | d-Oxy-Hb | -0.9 (-17.5, 15.7) | **32.0 (11.7, 52.2)*** | -17.7  (-40.1, 4.6) | 14.6  (-5.9, 35.1) | 21.6  (-1.6, 44.9) | 37.2  (9.2, 65.2) |
| **SRA** |  |  |  |  |  |  |  |
| POST-PRE | Oxy-Hb | 8.4 (-29.2, 46.1) | **-57.5 (-102.5, -12.4)*** | 22.0  (-25.6, 69.7) | -7.9  (-55.3, 39.5) | -27.7  (-81.9, 26.6) | -85.1  (-144.4, -25.8) |
|  | d-Oxy-Hb | -11.5 (-32.0, 8.9) | 2.4 (-18.3, 23.2) | -16.1  (-42.0, 9.9) | -8.9  (-36.1, 18.2) | -2.3  (-27.3, 22.7) | 7.2  (-21.0, 35.4) |
| Oxy-Hb, oxygenated hemoglobin; d-Oxy-­Hb, deoxygenated hemoglobin; CI, confidence interval; SOCIAL, social break condition; WALK, walking break condition; SRA, simple resistance activity break condition; PFC, prefrontal cortex. **q*-value ≤0.05 (FDR-adjusted p-values for multiple comparisons) for within condition changes post-pre changes. | | | | | | | |

| **Supplementary Table 5.** Arterial stiffness stratified changes in oxygenated- and deoxygenated-hemoglobin in the whole prefrontal cortex and in the left and right hemispheres, during the **3-back** cognitive test, and within condition (SOCIAL, WALK, SRA) changes from pre-test to post-test. | | | | | | | |
| --- | --- | --- | --- | --- | --- | --- | --- |
|  |  | **3-back**  Beta (95% confidence interval) | | | | | |
|  |  | WHOLE PFC | | High arterial stiffness | | Low arterial stiffness | |
| **Condition**/ Time | Parameter | High arterial stiffness | Low arterial stiffness | Left PFC | Right PFC | Left PFC | Right PFC |
| **SOCIAL** |  |  |  |  |  |  |  |
| PRE | Oxy-Hb | -20.5 (-49.1, 8.2) | -3.9 (-42.2, 34.3) | -19.2  (-54.0, 15.5) | -23.6  (-60.7, 13.5) | -26.0  (-71.2, 19.1) | 25.6  (-25.0, 76.3) |
|  | d-Oxy-Hb | -28.9 (-42.8, -15.0) | -9.3 (-25.2, 6.7) | -35.4  (-53.6, -17.2) | -21.8  (-39.7, -3.9) | -7.5  (-26.7, 11.8) | -13.2  (-34.4, 8.0) |
| POST | Oxy-Hb | -44.7 (-73.6, -15.8) | -38.8 (-69.7, -7.9) | -40.1  (-77.5, -2.8) | -42.6  (-79.3, -5.9) | -64.7  (-105.0, -24.5) | -13.6  (-51.7, 24.5) |
|  | d-Oxy-Hb | 0.8 (-15.6, 17.1) | 5.1 (-12.8, 23.0) | -0.6  (-22.0, 20.9) | 4.8  (-16.6, 26.3) | 9.5  (-12.6, 31.7) | 1.0  (-23.0, 25.0) |
| **WALK** |  |  |  |  |  |  |  |
| PRE | Oxy-Hb | -38.4 (-63.1, -13.7) | -55.0 (-93.4, -16.6) | -45.7  (-76.6, -14.8) | -36.6  (-68.5, -4.8) | -49.3  (-97.3, -1.4) | -38.7  (-87.9, 10.4) |
|  | d-Oxy-Hb | -16.8 (-30.3, -3.3) | -8.6 (-27.2, 10.0) | -20.1  (-38.3, -1.9) | -6.9  (-24.3, 10.6) | -20.4  (-41.9, 1.1) | 5.3  (-20.5, 31.1) |
| POST | Oxy-Hb | 16.6 (-4.4, 37.6) | 27.0 (3.6, 50.4) | -9.0  (-37.8, 19.7) | 15.4  (-8.6, 39.5) | 1.6  (-25.5, 28.7) | 51.1  (20.8, 81.4) |
|  | d-Oxy-Hb | -6.0 (-18.1, 6.1) | -22.9 (-34.0, -11.8) | 12.5  (-3.5, 28.6) | -8.5  (-23.0, 6.0) | -33.7  (-46.5, -20.9) | -15.0  (-30.1, 0.1) |
| **SRA** |  |  |  |  |  |  |  |
| PRE | Oxy-Hb | 6.8 (-19.9, 33.4) | -65.9 (-101.0, -30.7) | 8.5  (-24.8, 41.8) | 0.9  (-33.0, 34.7) | -55.7  (-97.6, -13.7) | -71.9  (-118.5, -25.3) |
|  | d-Oxy-Hb | -8.2 (-22.0, 5.7) | -33.9 (-50.0, -17.8) | -5.1  (-23.1, 12.9) | -9.0  (-27.0, 9.1) | -32.5  (-52.0, -13.1) | -28.9  (-50.6, -7.1) |
| POST | Oxy-Hb | -12.4 (-40.4, 15.6) | -22.7 (-53.0, 7.6) | -8.3  (-44.4, 27.9) | -15.5  (-50.4, 19.4) | -9.2  (-46.4, 28.0) | -38.2  (-77.4, 1.0) |
|  | d-Oxy-Hb | -24.5 (-40.8, -8.2) | -0.1 (-15.0, 14.7) | -14.5  (-35.2, 6.1) | -32.3  (-54.3, -10.4) | 1.3  (-16.9, 19.4) | -2.7  (-23.0, 17.5) |
| **WITHIN CONDITION POST-PRE CHANGES** | | | | | | | |
|  |  | **3-back**  Beta (95% confidence interval) | | | | | |
|  |  | WHOLE PFC | | High arterial stiffness | | Low arterial stiffness | |
| **Condition**/ Time | Parameter | High arterial stiffness | Low arterial stiffness | Left PFC | Right PFC | Left PFC | Right PFC |
| **SOCIAL** |  |  |  |  |  |  |  |
| POST-PRE | Oxy-Hb | 24.3 (-16.0, 64.5) | 34.9 (-13.9, 83.6) | 20.9 (-29.5, 71.3) | 19.0  (-32.5, 70.5) | 38.7  (-21.2, 98.6) | 39.3  (-23.5, 102.1) |
|  | d-Oxy-Hb | **-29.6 (-50.3, -8.9)*** | -14.4 (-37.5, 8.7) | -34.9 (-61.9, -7.8) | -26.6  (-53.4, 0.2) | -17.0  (-45.1, 11.1) | -14.2  (-45.1, 16.7) |
| **WALK** |  |  | | |  |  |  |
| POST-PRE | Oxy-Hb | **-55.0 (-86.9, -23.2)*** | **-82.0 (-126.5, -37.5)*** | -36.6 (-78.2, 5.0) | -52.1  (-91.1, -13.1) | -51.0  (-105.5, 3.6) | -89.8  (-146.9, -32.8) |
|  | d-Oxy-Hb | -10.8 (-28.0, 6.5) | 14.3 (-6.4, 35.0) | -32.6 (-55.8, -9.5) | 1.6  (-19.7, 22.9) | 13.3  (-10.3, 36.9) | 20.3  (-8.2, 48.9) |
| **SRA** |  |  |  |  |  |  |  |
| POST-PRE | Oxy-Hb | 19.1 (-19.1, 57.4) | -43.2 (-89.1, 2.8) | 16.7 (-31.8, 65.3) | 16.4  (-31.6, 64.4) | -46.5  (-101.9, 8.9) | -33.7  (-94.0, 26.6) |
|  | d-Oxy-Hb | 16.3 (-4.3, 37.0) | **-33.7 (-54.7, -12.8)*** | 9.4 (-16.8, 35.7) | 23.3  (-4.1, 50.8) | -33.8  (-59.1, -8.6) | -26.1 (-54.6, 2.4) |
| Oxy-Hb, oxygenated hemoglobin; d-Oxy-­Hb, deoxygenated hemoglobin; CI, confidence interval; SOCIAL, social break condition; WALK, walking break condition; SRA, simple resistance activity break condition; PFC, prefrontal cortex. **q*-value ≤0.05 (FDR-adjusted p-values for multiple comparisons) for within condition changes post-pre changes. | | | | | | | |

| **Supplementary Table 6.** Area under the curve for post-prandial glucose results during the 3-hr experimental conditions. | | | | | | | | | |
| --- | --- | --- | --- | --- | --- | --- | --- | --- | --- |
| Within conditions | | | Change between conditions | | | | | | |
| SOCIAL | WALK | SRA | WALK-SOCIAL | | SRA-SOCIAL | | WALK-SRA | | |
| Mean (SD) | Mean (SD) | Mean (SD) | Beta-Coefficient | P-value | Beta-Coefficient | P-value | Beta-Coefficient | P-value |  |
| 63.7 (6.4) | 61.1 (5.5) | 59.4 (7.4) | -2.5 | 0.2 | -3.8 | 0.1 | -1.3 | 0.5 |  |
| SOCIAL, social break condition; WALK, walking break condition; SRA, simple resistance activity break condition. SD, standard deviation. Missing 1 for conditions SOCIAL and SRA. | | | | | | | | | |

# Supplementary Figure 3

**Supplementary Figure 3.** Within condition, pre-test to post-test changes in 1-, 2-, and 3-back working memory tests’ accuracy and reaction time, stratified by arterial stiffness. SOCIAL, social break condition; WALK, walking break condition; SRA, simple resistance activity break condition.
